# Age- and sex-dependent changes in sympathetic activity of the left ventricular apex assessed by ^18^F-DOPA PET imaging

**DOI:** 10.1371/journal.pone.0202302

**Published:** 2018-08-14

**Authors:** Irene A. Burger, Christine Lohmann, Michael Messerli, Susan Bengs, Anton Becker, Monika Maredziak, Valerie Treyer, Achi Haider, Moritz Schwyzer, Dominik C. Benz, Ken Kudura, Michael Fiechter, Andreas A. Giannopoulos, Tobias A. Fuchs, Christoph Gräni, Aju P. Pazhenkottil, Oliver Gaemperli, Ronny R. Buechel, Philipp A. Kaufmann, Catherine Gebhard

**Affiliations:** 1 Department of Nuclear Medicine, University Hospital Zurich, Zurich, Switzerland; 2 Department of Diagnostic and Interventional Radiology, University Hospital Zurich, Zurich, Switzerland; 3 Center for Molecular Cardiology, University of Zurich, Zurich, Switzerland; University of Messina, ITALY

## Abstract

**Background:**

Sexual dimorphism in cardiac sympathetic outflow has recently gained attention in the context of Takotsubo cardiomyopathy. Previous studies suggest that there are sex- and age-dependent differences in peripheral autonomic control, however, data on cardiac-specific sympathetic activation in aged women and men are lacking.

**Methods and results:**

Regional quantitative analysis of cardiac fluorine-18 (^18^F)- Dihydroxyphenylalanine (DOPA) uptake was retrospectively performed in 133 patients (69 females, mean age 52.4±17.7 years) referred for assessment of neuroendocrine tumours (NET) by Positron-Emission-Tomography. Cardiac ^18^F-DOPA uptake was significantly higher in women as compared to men (1.33±0.21 vs. 1.18±0.24, p<0.001). This sex-difference was most pronounced in the apical region of the left ventricle (LV, 1.30±0.24 in women vs. 1.13±0.25 in men, p<0.001) and in individuals >55 years of age (1.39±0.25 in women vs. 1.09±0.24 in men, p<0.001). Women showed a prominent increase in myocardial ^18^F-DOPA uptake with age with the strongest increase seen in the LV apical region (r = 0.34, p = 0.004). Accordingly, sex and age were selected as significant predictors of LV apical ^18^F-DOPA uptake in a stepwise linear regression model. No age-dependent changes of cardiac ^18^F-DOPA uptake were observed in men or in the right ventricular region.

**Conclusion:**

Our study suggests that aging is related to sex-specific changes in regional cardiac sympathetic activity. Future studies will have to assess whether the increase in LV apical ^18^F-DOPA uptake with age in women is of pathogenic relevance for the higher susceptibility of postmenopausal women to conditions associated with increased sympathetic activity.

## Introduction

Cardiovascular disease is the leading cause of death and disease burden in both, women and men, in the western world. While cardiovascular mortality rates have rapidly declined since the late 1970s in men, death rates in women have not improved to the same extend. In fact, the disease is becoming more common in women, in particular in young women, and cardiovascular deaths in women currently exceed those in men.[1, 2] However, there is currently only limited data on sex-specific pathogenesis, management, and outcomes of cardiovascular disease.

Sexual dimorphism in cardiac sympathetic outflow has recently gained increasing attention in the context of Takotsubo cardiomyopathy or cardiac syndrome X.[3–6] The worse outcomes observed in women with cardiovascular disease as well as their higher susceptibility to cardiac injury during high-stress situations implies that sex-differences in autonomous nervous control of the cardiovascular system might be pathogenetic.[7, 8] However, there is a lack of data on sex- and age-specific cardiac sympathetic activity in normal individuals and very little information about regional norepinephrine turnover, uptake, and metabolism in the human myocardium is available in the literature. Indeed, previous quantification of sympathetic activity in humans was mainly obtained by unspecific approaches such as power spectral analysis of heart rate variability (HRV), measurement of muscle sympathetic nervous activity (MSNA) or quantification of circulating catecholamine levels and have produced widely varying results.[9, 10] Notably, while MSNA seems to be a good indicator of cardiac or renal sympathetic activity and vasoconstrictor tone in men, studies investigating this association in women are sparse and have failed to detect a significant correlation between MSNA and cardiac output or vasoconstrictor tone.[11]

Measurement of regional cardiac sympathetic activity by sympathetic neurotransmitter radionuclide analogues (e.g. ^123^I-Metaiodobenzylguanidine [MIBG]) has been shown to add incremental prognostic value in predicting disease progression in heart failure patients beyond that provided by traditional markers.[12] Hence, regional dysfunction of the sympathetic nervous system might add important prognostic information about cardiac vulnerability of women and men and may help to direct preventive strategies and therapy. Besides ^123^I-MIBG for scintigraphy, an ^18^F labelled Positron-Emission-Tomography (PET) tracer was developed using the catecholamine precursor fluorine-18 (^18^F)-Dihydroxyphenylalanine (^18^F-DOPA) to image active paragangliomas, tumours derived from the autonomous nervous system.[13]

Given the different susceptibility of postmenopausal women and older men to cardiac conditions associated with sympathetic dysregulation, we hypothesized that sex- and age-dependant differences exist with regard to cardiac autonomic control. As the superior spatial resolution of PET allows to assess even small differences in tracer uptake, the primary aim of our study was the quantification of regional cardiac ^18^F-DOPA uptake in men and women free of cardiovascular disease at different ages.

## Methods

### Study population

Cardiac uptake of ^18^F-DOPA was retrospectively assessed in 133 individual patients (69 females, mean age 52.4±17.7 years, range 1–84 years) who underwent functional imaging with ^18^F-DOPA PET-CT for evaluation of a known or suspected NET at our institution between February 2007 and March 2016. Repeated ^18^F-DOPA exams were excluded from our study. Information on medical history and cardiovascular risk factors (hypertension, hyperlipidemia, diabetes, smoking, family history of premature CAD) were obtained from electronic patient records. Subjects with structural heart disease including heart failure and valvular heart disease, known obstructive coronary artery disease, previous myocardial infarction, previous coronary artery bypass grafting or percutaneous coronary intervention, hypertension or diabetes mellitus were excluded from our analysis. The study conforms to the principles outlined in the 1964 Declaration of Helsinki and was approved by the local cantonal ethics committee in Zurich, Switzerland (BASEC No. 2017–01112). The need to obtain informed consent was waived by the ethics committee due to the retrospective nature of the study.

### Image acquisition and reconstruction

DOPA is a neutral amino acid that resembles natural L-DOPA (dopamine precursor). It enters the catecholamine metabolic pathway of endogenous L-DOPA in the brain and peripheral tissues and can be labeled with ^18^F (half-life 110 min) for PET imaging. Incerased uptake of ^18^F-DOPA is seen in tissues with high activities of L-DOPA decarboxylase. ^18^F-DOPA-PET was used at our institution after its formal approval for use in Europe (November 2006). Patients were asked to fast for at least 4–6 h before ^18^F-DOPA injection. ^18^F-DOPA-PET was performed 45 min after injection ^18^F-DOPA (mean 202.8±36.7 MBq, range 25–263 MBq) into a peripheral vein.[14] Images were acquired in 3D mode on different scanners (Discovery VCT or Discovery RX (GE-Healthcare, Milwaukee, WI, USA). The imaging protocol consisted of a scout view followed by low-dose CT acquisition for attenuation correction and subsequent PET acquisition. PET emission scans were acquired from the base of the skull to mid-thigh during 20 min (5–7 bed positions of 2–3 min each). Iterative reconstruction and CT-based attenuation correction were used. PET and CT images were fused using a dedicated software package (AW 5.0 GE-Healthcare). PET scans were acquired without premedication with carbidopa.

### Data analysis

Reconstructed data were transferred to an external workstation (Advantage AW 4.4, GE Healthcare) for analysis. On reformatted horizontal long-axis slices, the left ventricle (LV) and right ventricle (RV) were subdivided into a total of six segments (LV basal and midventricular lateral wall, LV basal and midventricular septum, LV septal and lateral apical segments, and RV basal and midventricular lateral wall). Cardiac segments in our study were defined based on the recommendations of the American Heart Association Writing Group on Myocardial Segmentation and Registration for Cardiac Imaging.[15] A semiquantitative analysis of regional ^18^F-DOPA uptake was performed by placing a circular volume of interest (VOI) of 2.3cm^3^, automatically generated by the computer, into these four segments. The size of the VOI was standardized for all images by using a semi-automatic volume analysis tool (GE Healthcare, Milwaukee, USA). A semiquantitative uptake analysis using the upper limit of the standardized uptake value (SUV_max_) and the averaged standardized uptake value (SUV_mean_) was used to quantify ^18^F-DOPA uptake in these regions. SUV_max_ and SUV_mean_ were normalized to blood pool ^18^F-DOPA activity measured in the aortic arch of each patient (= SUV_max-N_ and SUV_mean-N_).

### Statistical analysis

Data are presented as mean ± standard deviation (SD) for continuous variables and frequency and percentage for categorical variables. Data were stratified for sex and age. The age of 55 years was used as a cut-off value to differentiate between younger and older individuals. This cut-off value was chosen given that most women undergo menopause between 45 and 55 years and that, at the age of 55 years, 95% of women are postmenopausal.[16] Prior to analyses, basic assumptions were checked. Student’s t-test, Mann-Whitney test, analysis of variance (ANOVA) or Kruskal-Wallis test were used for group comparisons of continuous variables. For comparison of different age- and sex-groups, p-values were adjusted by the Bonferroni correction for multiple tests. For categorical variables, chi-square tests or Fisher's exact test were used, as appropriate. Multivariate linear regression analysis was applied to assess the association of age and sex with regional cardiac sympathetic activity. All tests were two-sided, and *p* values below 0.05 were considered significant. Statistical analyses were performed with IBM SPSS statistics v24.0 and GraphPad Prism (v4.0, GraphPad Software, San Diego, CA).

## Results

### Patients characteristics

Regional quantitative analysis of cardiac ^18^F-DOPA uptake was performed in reconstructed PET/CT images of all 133 patients. All patients were specifically referred for ^18^F-DOPA PET-computed tomography (CT) for evaluation of a known or suspected pheochromocytoma (n = 26), carcinoid (n = 15), thyroid carcinoma (n = 18), or extra-adrenal paraganglioma (n = 60, Table 1) according to clinical, biochemical or radiological data. In two cases the reason for ^18^F-DOPA PET imaging was the presence of a carcinoma of unknown primary origin, two patients were referred for evaluation of ectopic adrenocorticotropic hormone production and 10 cases had suspicious hepatic, pancreatic and adrenal masses (Table 1). In 67 (50.4%) patients ^18^F-DOPA scans were positive for paraganglioma. When data were stratified by sex, no significant differences with regard to baseline characteristics were observed between men and women (p = NS, Table 1), except for BMI, which was significantly higher in men (p<0.001, Table 1). Clinical indications for ^18^F-DOPA PET referral and patients characteristics stratified by sex are depicted in Table 1.

**Table 1:** Patient baseline, ^18^F-DOPA PET acquisition characteristics, regional cardiac ^18^F-DOPA uptake, and clinical indications for ^18^F-DOPA PET imaging.

Baseline characteristics	Total n = 133	Womenn = 69	Menn = 64	p-value
**Age (years), mean±SD**	52.4±17.7	52.9±19.2	52.0±16.1	0.8
**BMI, mean±SD**	22.7±6.8	20.4±5.4	25.2±7.2	<0.001
^**18**^ **F-DOPA positive scan, n(%)**	67(50.4)	31(44.9)	36(56.3)	0.2
**Injected** ^**18**^**F-DOPA dose (MBq), mean±SD**	202.8±36.7	204.4±40.1	201.2±32.9	0.6
**Reason for referral, n(%)**				0.3
Clinical suspicion of carcinoid	5(3.8)	2(2.9)	3(4.7)	
Clinical suspicion of pheochromocytoma	13(9.8)	8(11.6)	5(7.9)	
Clinical suspicion of extra-adrenal paraganglioma	13(9.8)	6(8.7)	7(11.1)	
Treatment control carcinoid	10(7.5)	7(10.1)	3(4.7)	
Treatment control thyroid carcinoma	18(13.5)	9(13.0)	9(14.3)	
Treatment control pheochromocytoma	13(9.8)	5(7.2)	8(12.7)	
Treatment control extra-adrenal paraganglioma	47(35.3)	26(37.7)	21(33.3)	
Carcinoma of unknown primary origin	2(1.5)	1(1.4)	1(1.5)	
Ectopic ACTH production	2(1.5)	0(0)	2(3.2)	
Suspicious mass	10(7.5)	6(8.7)	4(6.3)	
**Medical treatment**				
Antiadrenergic compounds	26(19.5)	17(24.6)	9(14.1)	0.12
Immunosuppressive/antiinflammatory agents	13(9.8)	7(10.1)	6(9.4)	0.88
Somatostatin analogues	16(12)	8(11.6)	8(12.5)	0.87
Thyroid hormone receptor agonists	18(13.5)	10(14.5)	8(12.5)	0.74
**Total myocardial** ^**18**^**F-DOPA uptake (SUV**_**max-N**_**), mean±SD**	1.26±0.24	1.33±0.21	1.18±0.24	<0.001
**Total myocardial** ^**18**^**F-DOPA uptake (SUV**_**mean-N**_**), mean±SD**	0.86±0.17	0.94±0.15	0.78±0.14	<0.001
**Total LV** ^**18**^**F-DOPA uptake (SUV**_**max-N**_**), mean±SD**	1.31±0.26	1.39±0.24	1.23±0.26	<0.001
**Total LV** ^**18**^**F-DOPA uptake (SUV**_**mean-N**_**), mean±SD**	0.90±0.18	0.98±0.17	0.80±0.15	<0.001
**LV-mid-ventricular** ^**18**^**F-DOPA uptake (SUV**_**max-N**_**), mean±SD**	1.32±0.25	1.39±0.24	1.25±0.25	<0.001
**LV-mid-ventricular** ^**18**^**F-DOPA uptake (SUV**_**mean-N**_**), mean±SD**	0.91±0.17	0.99±0.15	0.83±0.14	0.001
**RV** ^**18**^**F-DOPA uptake (SUV**_**max-N**_**), mean±SD**	1.09±0.21	1.14±0.20	1.02±0.20	0.001
**RV** ^**18**^**F-DOPA uptake (SUV**_**mean-N**_**), mean±SD**	0.77±0.16	0.82±0.12	0.71±0.17	<0.001
**LV-apical** ^**18**^**F-DOPA uptake (SUV**_**max-N**_**), mean±SD**	1.28±0.28	1.30±0.24	1.13±0.25	<0.001
**LV-apical** ^**18**^**F-DOPA uptake (SUV**_**mean-N**_**), mean±SD**	0.88±0.19	0.94±0.18	0.75±0.16	<0.001

NET, neuroendocrine tumor; ACTH, adrenocorticotropic hormone; SUV_max-N_, upper limit of the standardized uptake value normalized to blood pool; SUV_mean-N_, averaged standardized uptake value normalized to blood pool; RV right ventricular; LV, left ventricular. Values are indicated as mean±standard deviation (SD) or n(%). P-values are indicated for women vs men.

### Overall cardiac ^18^F-DOPA uptake in women and men

As previously reported, mild ^18^F-DOPA uptake is apparent in the myocardium, peripheral muscles, esophagus, and in some cases in the mammary glands.[14] Overall cardiac ^18^F-DOPA uptake was significantly higher in women as compared to men (p<0.001, Table 1) and increased significantly with age in women (Pearson r = 0.32, p = 0.008) but not in men (Pearson r = -0.003, p = 0.97, data not shown). When overall cardiac ^18^F-DOPA uptake was measured in both, ^18^F-DOPA-negative for NET (n = 66) and ^18^F-DOPA-positive for NET (n = 67) patients, no differences in myocardial ^18^F-DOPA uptake were found (p = NS, data not shown). The latter was true for both, men and women (p = NS, data not shown). Similarly, when patients were stratified by medical treatment including antiadrenergic therapy, immunosuppressive/anti-inflammatory agents, somatostatin analogues, and thyroid hormone receptor agonists, no significant differences in myocardial ^18^F-DOPA activity were found in patients with and without treatment (1.30±0.23 vs 1.25±0.24 SUV_max-N_, p = 0.37 for antiadrenergic therapy; 1.29±0.29 vs 1.25±0.23 SUV_max-N_, p = 0.46 for somatostatin analogues; 1.26±0.24 vs 1.27±0.18 SUV_max-N_, p = 0.35 for immunosuppressive/anti-inflammatory agents; and 1.26±0.25 vs 1.24±0.17 SUV_max-N_, p = 0.76 for thyroid hormone receptor agonists).

### Sex- and age-dependent changes in regional cardiac ^18^F-DOPA uptake

**Fig 1:**
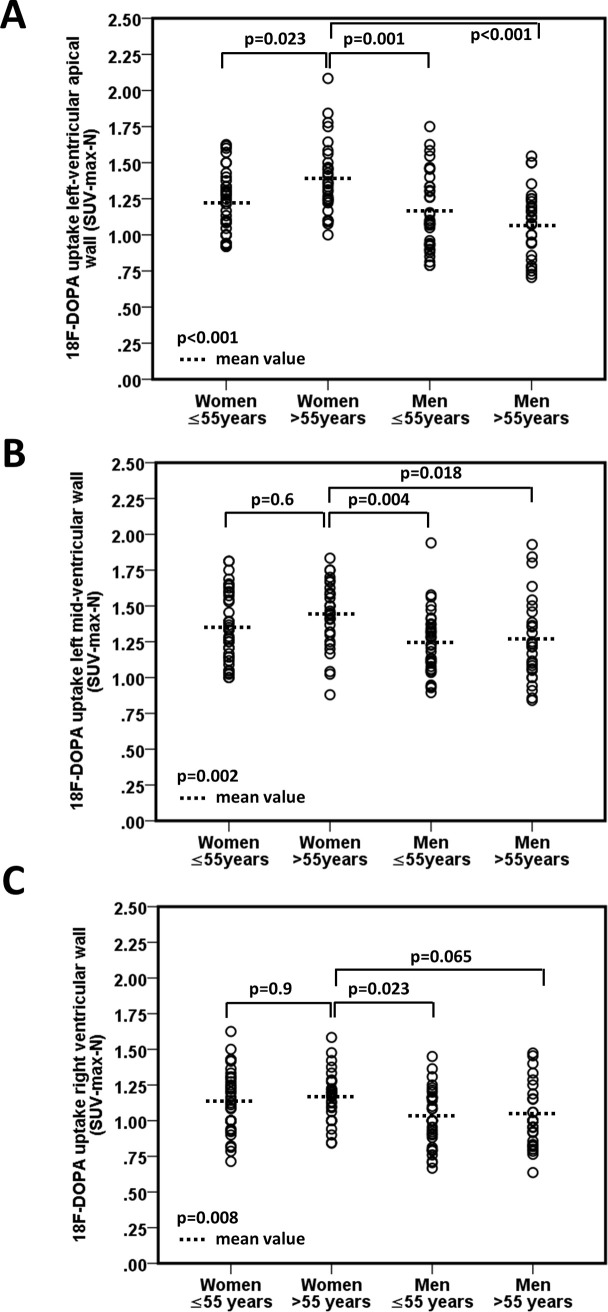
Comparative analysis of myocardial ^18^F-DOPA uptake stratified by age (<55 years and >55 years) and sex. ^18^F-DOPA uptake was measured in the apical **(A)**, left mid-ventricular **(B)**, and right ventricular **(C)** region of the heart. Data are presented as dot plots. Mean values and p-values (overall and post-hoc tests) are indicated.

**Fig 2:**
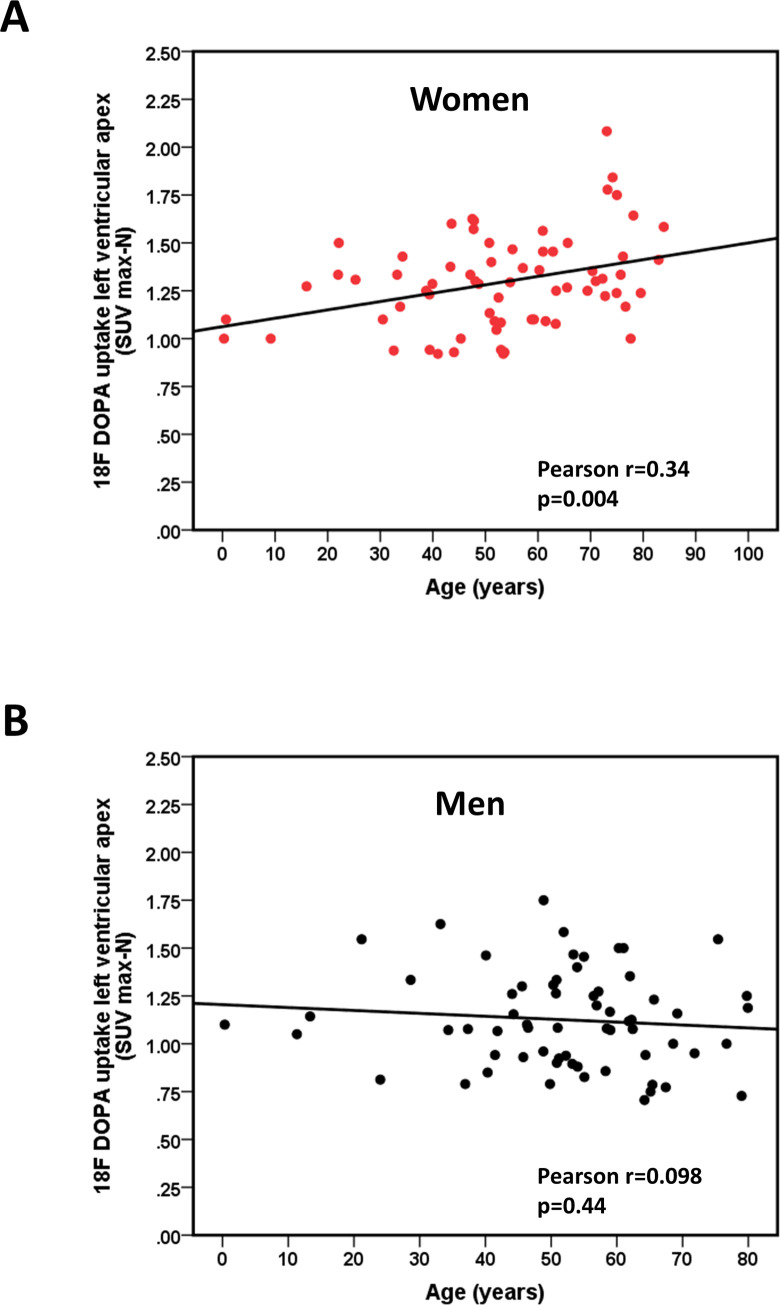
Age-dependent increase in ^18^F-DOPA uptake in women. **A.** Correlation between age and left ventricular apical ^18^F-DOPA uptake (SUV_max-N_) in women. Pearson correlation coefficients and p-values are indicated. **B.** Correlation between age and left ventricular apical ^18^F-DOPA uptake (SUV_max-N_) in men. Pearson correlation coefficients and p-values are indicated. Pearson correlation coefficients and p-values are indicated.

**Fig 3:**
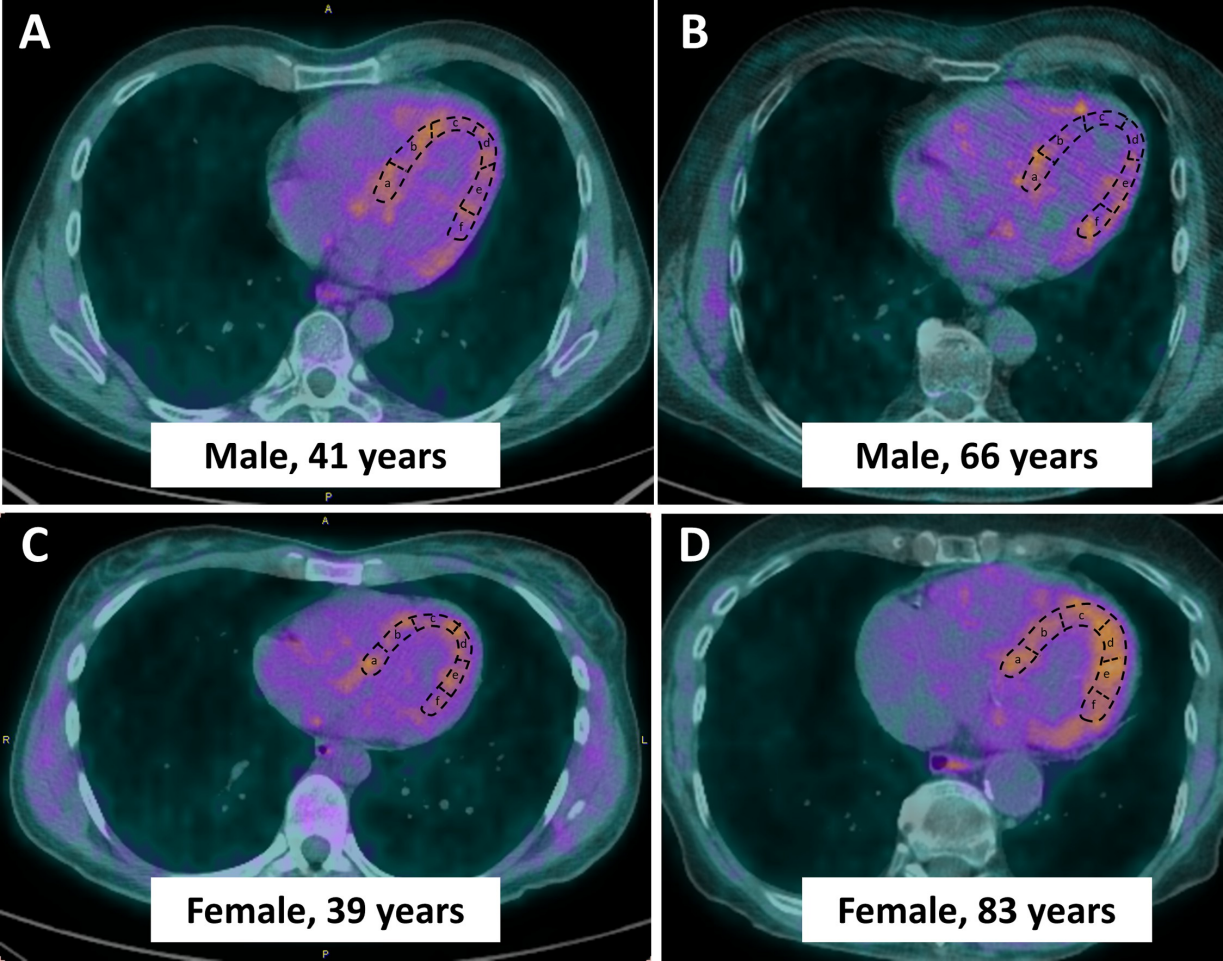
Example of Positron-Emission-Tomography image reconstruction using the pmod cardiac PET modelling tool (pmod version 3.8, PMOD technologies LLC, Zurich, Switzerland) and quantification of ^18^F-DOPA uptake in the left ventricular wall. A semiquantitative uptake analysis using the upper limit of the standardized uptake value (SUV_max-N_) and the mean SUV (SUV_mean-N_) at the sites of physiologic uptake was performed using a planar circular region of interest of 1 cm diameter, automatically generated by the computer. Left ventricular segments in the horizontal long axis were defined as follows: **a** = basal septal, **b** = midventricular septal, **c** = apical septal, **d** = apical lateral, **e** = midventricular lateral, **f** = basal lateral. **A.** Cardiac long axis image of a 41 year old male patient. **B.** Cardiac long axis image of a 66 year old male patient. **C.** Cardiac long axis image of a 39 year old female patient. **D.** Cardiac long axis image of a 72 year old female patient. Similar thresholds were applied for all images.

When patients were stratified by sex and age (≤ 55 and > 55 years), women >55 years had the highest cardiac ^18^F-DOPA uptake (p<0.05, ANOVA, Fig 1A–1C). ^18^F-DOPA uptake in the LV apical region increased significantly with age in women (p = 0.025 for women >55 years vs women ≤55 years, Fig 1A), while sex- and age differences in ^18^F-DOPA uptake were less pronounced in the left midventricular region (Fig 1B) and absent in the RV (Fig 1C). No age-dependent changes in cardiac ^18^F-DOPA uptake were observed in men (Fig 1A–1C). When a sub-analysis in patients >70 years was performed (n = 17 for women and n = 6 for men), we observed a further increase in LV apical ^18^F-DOPA uptake in women >70 years, while no such tendency was seen in older men or in other LV regions. In detail, apical ^18^F-DOPA uptake was 1.5±0.3 SUV_max-N_ in women >70 years, 1.3±0.2 SUV_max-N_ in women >55 and ≤70 years, and 1.2±0.2 SUV_max-N_ in women ≤55 years (p = 0.003). Conversely, no changes in LV apical ^18^F-DOPA uptake were observed in men >70 years as compared to younger age groups (1.1±0.3 SUV_max-N_ in men >70 years, 1.1±0.3 SUV_max-N_ in men >55 and ≤70 years, and 1.2±0.3 SUV_max-N_ in men ≤55 years (p = 0.5). Hence, a positive and significant correlation between age and LV apical ^18^F-DOPA uptake was seen in women (Fig 2A), while age and LV apical ^18^F-DOPA uptake were not associated in men (Fig 2B). In contrast, left mid-ventricular ^18^F-DOPA uptake was not associated with age in either sex (r = 0.2, p = 0.083 in women and r = 0.03, p = 0.8 in men, data not shown). Similar, no age-dependent changes of cardiac ^18^F-DOPA uptake were observed in the RV (p = NS, data not shown). Fig 3 shows representative examples of reconstructed cardiac long axis ^18^F-DOPA-PET-CT images of all sex and age groups.

### Predictors of regional cardiac sympathetic activity

When sex and age were tested in a stepwise linear regression analysis with LV apical and mid-ventricular ^18^F-DOPA uptake being the dependent variable, and BMI and pathologic findings as predictor variables, both, sex and age were identified as significant predictors for LV apical ^18^F-DOPA uptake (Table 2). The probability was best explained by sex, followed by age (Table 2). When predictors for left mid-ventricular ^18^F-DOPA uptake were tested, sex remained a significant predictor for left mid-ventricular ^18^F-DOPA uptake, while age was not selected by this model (Table 3).

**Table 2:** Stepwise linear regression model for left-ventricular apical ^18^F DOPA uptake (n = 133).

Independent variables	B coefficient (SE)	p-value
Male Sex	-0.15 (0.045)	0.003
Age	0.004 (0.001)	0.005

Stepwise method was performed among age, sex, body mass index (BMI) and pathologic findings on ^18^F-DOPA scan. Only variables staying in the final model are presented. SE, standard error.

**Table 3:** Stepwise linear regression model for left mid-ventricular ^18^F DOPA uptake (n = 133).

Independent variables	B coefficient (SE)	p-value
Male Sex	-0.11 (0.047)	0.01

Stepwise method was performed among age, sex, body mass index (BMI) and pathologic findings on ^18^F-DOPA scan. Only variables staying in the final model are presented. SE, standard error.

## Discussion

In this retrospective single centre study, we performed a sex- and age-specific analysis of regional cardiac sympathetic activity in patients undergoing ^18^F-DOPA PET-CT for evaluation of NET. Significantly higher cardiac ^18^F-DOPA uptake was observed in women as compared to men. This sex-difference in ^18^F-DOPA uptake was most pronounced in the apical region of the LV. With advancing age, ^18^F-DOPA uptake significantly increased in women, in particular in the LV apex, while no age-dependent changes of ^18^F-DOPA uptake were observed in men.

While physiological uptake of ^18^F-DOPA in extraneuronal tissue including liver, myocardium and peripheral muscles has been described previously [14, 17], our study is the first to report sex-and age-specific differences in regional myocardial ^18^F-DOPA uptake in patients without obvious cardiovascular disease. Given its role as precursor of the neurotransmitters dopamine, epinephrine, and norepinephrine, enhanced ^18^F-DOPA uptake mirrors an increased activity of L-DOPA decarboxylase.[17] The latter is associated with an increased synthesis and turnover of norepinephrine in the adrenergic nerve terminals, thereby signifying an increase in sympathetic outflow. Accordingly, enhanced cardiac L-DOPA decarboxylase activities have been associated with increased conversion of [3H]tyrosine to [3H]catecholamine as well as with a faster metabolic rate of norepinephrine in diabetic rats.[18] Therefore, our observations suggest a sympathetic dominance in the LV apical region which is progressively prominent with aging in women but not in men. While our observations are consistent with earlier studies reporting an increase in systemic sympathetic drive assessed by MSNA or norepinephrine spillover in older subjects, previous MIBG studies have failed to report consistent data on age- and sex-specific cardiac sympathetic activity.[9, 19, 20] In fact, while Sakata et al. observed a gradual decrease of ^123^I-MIBG uptake alongside an increase in ^123^I-MIBG washout rate with age in both sexes, Tsuchimochi et al. did not observe any differences in MIBG uptake based on age or sex.[21, 22] Heterogenous cohorts, small sample sizes as well as differences in acquisition protocols might have accounted for these discrepancies. In addition, according to our data, age-dependent changes in LV innervation seem not to be uniform and previous data might be inconsistent due to the assumption that the sympathetic nervous system in the human LV would change homogeneously with age.

While our study clearly demonstrates physiologic changes in regional cardiac sympathetic activity with age, the mechanisms underlying this age-dependent remodeling of cardiac sympathetic outflow remain unclear. Among the postulated mechanisms, age-dependent changes in hormone levels are notable as well as attenuation of parasympathetic activity in postmenopausal women.[23] Indeed, several studies have reported an inhibiting effect of hormone replacement therapy on sympathetic activity in postmenopausal women, while lower testosterone levels have been associated with increased sympathetic excitability in older men with cardiac hypertrophy.[24–26] In addition, an aging-related decline in norepinephrine transporter/uptake-1 activity has been suggested to enhance the delivery of catecholamines to postsynaptic sites.[27] Finally, the observed increase of apical ^18^F-DOPA uptake in women >55 years of age could be due to a denser sympathetic innervation as a result of an age-dependent remodeling of the female heart. Indeed, a stronger effect of age on LV size and function has been observed in women as compared to men as recent observational studies have reported a higher percentage of small hearts alongside a higher LV ejection fraction in aged females as compared to younger women and age-matched men.[28]

Although cardiac sympathetic function is adversely altered in many disease states, a causal relationship between the observed increase in sympathetic activity in postmenopausal females and their enhanced cardiac vulnerability remains to be established. However, it is known that during an acute coronary syndrome (ACS), women developed a relative greater magnitude of sympathetic activation than men which lasts until its resolution at nine months.[29] The latter is consistent with reports of a worse prognosis in women observed during this time period.[30] In addition, an enhanced vascular transduction of sympathetic activity into hemodynamic parameters due to a decrease in β-adrenergic vasodilatation has been postulated in older women. [31–33] Thus, unopposed α-adrenergic vasoconstriction along with enhanced sympathetic activity might predispose aged females to cardiac susceptibility in high-stress situations. Indeed, cardiac diseases associated with an augmented neural response to mental stress, such as Takotsubo cardiomyopathy, are highly prevalent in postmenopausal females.[3] Notably, the distribution pattern of myocardial ^18^F-DOPA uptake in women >55 years of age mirrors the distribution of LV dysfunction in Takotsubo cardiomyopathy which is least in the LV base and worst in the cardiac apex, resulting in apical ballooning. Similarly, an epinephrine-dependent switch from beta(2)-adrenoceptor-G(i) to G(s) protein signalling with subsequent negative inotropic effect in stress-induced cardiomyopathy, has been shown to be greatest at the apical myocardium.[34]

In light of the strong association between disease state and sympathetic dysregulation, our data suggest that the quantification of cardiac sympathetic outflow might add prognostic information about cardiac vulnerability in women beyond that provided by traditional risk factors. Indeed, there is accumulating evidence that imaging of cardiac sympathetic activity may help to direct clinical decision making in patients with heart failure, a condition characterized by dysfunction of the sympathetic nervous system.[12] As increased sympathetic tone can be targeted pharmacologically and non-pharmacologically, early identification of patients at risk might offer the possibility to select appropriate preventive strategies and tailor therapeutic approaches. However, despite its FDA approval for heart failure patients, the exact role of cardiac neuronal imaging is still under debate, and, therefore sympathetic imaging modalities such as ^123^I-Metaiodobenzylguanidine (*m*IBG) scintigraphy or ^11^C-mHED PET are still mostly applied as a research method.[12, 35–37] Nevertheless, the limited prognostic value of current diagnostic strategies in women,[38–41] their overall higher cardiovascular mortality, and the increasing prevalence of cardiovascular disease due to the ageing of the population emphasize the need to better identify and understand the substrate that places postmenopausal women at risk. Further research is needed to assess whether sex-differences in cardiac sympathetic activity may account for the higher cardiovascular mortality seen in women and the different outcomes observed in pre- and post-menopausal women. In addition, larger-scale investigations are warranted to delineate whether regional cardiac sympathetic activity adds an incremental prognostic value in predicting cardiovascular risk beyond that provided by traditional functional and neurohormonal markers.

As with any study, certain design limitations are inherent. First, this study is a retrospective analysis from a single center, which limits its generalizability. Second, no follow-up data were available from our study cohort, thus, no conclusions can be made regarding an association between regional cardiac sympathetic activity and the risk of future adverse cardiovascular events. Third, no standardized dose and timing of ^18^F-DOPA PET currently exists and the ^18^F-DOPA activity administered varies largely in the literature.[17] However, the 45 min time interval between injection and acquisition as well as the administered dose of 202.8±36.7 MBq in our study lies within in the range of doses and timing reported in recent studies and has been recommended by the 2012 European Association of Nuclear Medicine (EANM) guidelines.[42] Fourth, unspecific uptake of radiolabeled amino acids in inflamed tissue, scar tissue, irradiated or ischemic areas has been reported and cannot be excluded in the present study. However, given that our study subjects did not have abnormalities suspicious of heart disease, it is unlikely that ischemic or inflammatory cardiac processes account for our findings. Finally, although no difference in cardiac ^18^F-DOPA uptake between ^18^F-DOPA positive and negative scans were observed, an effect of NET on cardiac DOPA decarboxylase activity cannot be ruled out in our study. Similarly, as myocardial perfusion imaging has not been performed in our study and despite the normalization of myocardial uptake to blood pool ^18^F-DOPA activity, an effect of myocardial blood flow on tracer kinetics cannot be completely excluded.

Taken together, our study suggests that a sympathetic dominance in the LV apex becomes progressively prominent with aging in women but not in men. Our data point out that understanding the multifactorial nature of cardiac autonomic modulation may provide diagnostic insights for a sex-specific cardiovascular disease management. Such a personalized approach will become ever more important in the upcoming years, given the ageing of the population and the increasing burden of environmental stress and cardiovascular disease. Future studies will have to evaluate whether there is a clinical role for cardiac neuronal imaging in phenotyping patients at risk for future cardiac events.

## Supporting information

S1 Table Anonymized dataset. Normalized myocardial ^18^F DOPA uptake per patient.
(XLSX)
